# Reply to Mrakic-Sposta et al. Comment on “Menzel et al. Common and Novel Markers for Measuring Inflammation and Oxidative Stress Ex Vivo in Research and Clinical Practice—Which to Use Regarding Disease Outcomes? *Antioxidants* 2021, *10*, 414”

**DOI:** 10.3390/antiox10060865

**Published:** 2021-05-28

**Authors:** Alain Menzel, Hanen Samouda, Francois Dohet, Suva Loap, Mohammed S. Ellulu, Torsten Bohn

**Affiliations:** 1 Laboratoires Réunis, 38, Rue Hiehl, L-6131 Junglinster, Luxembourg; amenzel@pt.lu (A.M.); dnalliance@gmail.com (F.D.); 2 Nutrition and Health Research Group, Department of Population Health, Luxembourg Institute of Health, 1 A–B, Rue Thomas Edison, L-1445 Strassen, Luxembourg; hanene.samouda@lih.lu; 3 Clinic Cryo Esthetic, 11 Rue Éblé, 75007 Paris, France; suvaloap@gmail.com; 4 Department of Clinical Nutrition, Faculty of Applied Medical Sciences, Al-Azhar University of Gaza (AUG), Gaza City 00970, Palestine; mohdsubhilulu@gmail.com

We appreciate the commentary by Mrakic-Sposta et al. on our published article [1]. We apologize if the erroneous picture was conveyed that electron paramagnetic resonance (EPR) has no clinical application and cannot, due to technical limitations, be used to obtain valuable data on reactive oxygen species (ROS) that can be linked to chronic diseases. Our intention was to point out that this sophisticated technique tends to employ more instrumentation and requires very careful handling and interpretation, and that for this reason it has been less frequently used in research and in clinical studies compared to the more “classical” endpoints. Indeed, EPR has been used in clinical studies, though most of these are of small or mid-sized scale as opposed to the many large-scale ones focusing on chronic disease, with often several thousand participants.

We are also glad about the further input regarding the capability of the technique to be applied to frozen/stored samples, including frozen saliva [2]. Indeed, the use of frozen samples has been reported also by others [3], and we admit that we originally were unaware of this. However, it was also emphasized that many ROS and reactive nitrogen species are very short-lived, posing a challenge for the accurate assessment of such species, and thus real-time measurements may be preferred. We also agree that EPR has been increasingly used for a number of biological matrices (blood, urine, saliva) and has indeed been employed for measuring NO [4], O_2_**^−^** and ·OH [5] and antioxidant capacity (based on copper reduction [6]), and that EPR results correlated well with protein carbonyls [7], or ROS, as measured by HPLC [8]. Thus, we fully agree with the authors of the commentary and look forward to hearing more about this promising technique in the near future and hope that EPR will see further use in clinical studies. 
